# The prevalence of the term subluxation in North American English-Language Doctor of chiropractic programs

**DOI:** 10.1186/2045-709X-19-14

**Published:** 2011-06-17

**Authors:** Timothy A Mirtz, Stephen M Perle

**Affiliations:** 1 Indiana Institute of Technology, School of Education, Fort Wayne, Indiana USA; 2 University of Bridgeport College of Chiropractic, Bridgeport, Connecticut, USA; 3 School of Chiropractic and Sports Science, Murdoch University, Murdoch, Western Australia, Australia

## Abstract

**Background:**

The subluxation construct has been a divisive term in the chiropractic profession. There is a paucity of evidence to document the subluxation. Some authors have questioned the propriety of continuing to use the term.

**Aim:**

The purpose of this study is to examine current North American English language chiropractic college academic catalogs and determine the prevalence of the term subluxation in the respective chiropractic program curricula.

**Methods:**

Sixteen current English-language North American chiropractic college academic catalogs were studied. The term subluxation was searched for in each of the catalogs. Categories were developed for the usage of the term. These included "total times mentioned", "subluxation mentioned in a course description", "subluxation mentioned in a course title", "subluxation mentioned in a technique course description", and "subluxation mentioned in a philosophy course description." The prevalence of the "subluxation mentioned in a course description" was compared to the total programmatic curriculum.

**Results:**

Palmer College in Florida devoted 22.72% of its curriculum to courses mentioning the subluxation followed by Life University (Marietta, GA) and Sherman College with 16.44% and 12.80% respectively. As per specific coursework or subjects, an average of 5.22 courses or subjects have descriptions mentioning the term subluxation. Three schools made no mention of the term subluxation in their academic catalogs; they were National University of Health Sciences, Canadian Memorial Chiropractic College, and Southern California University of Health Sciences.

**Conclusion:**

Despite the controversies and paucity of evidence the term subluxation is still found often within the chiropractic curricula of most North American chiropractic programs. Future research should determine if changes in accreditation standards and research on evidence based practice will affect this prevalence.

## Introduction

Chiropractic educational institutions are in expectation of approaching a high level of academic maturity [1]. Defining academic maturity is a challenging proposition. The authors of this paper define academic maturity by adapting Engelbrecht and Harding's definition [2]. Academic maturity as it applies to institutions means that institutions take responsibility for their own learning and that these institutions can make responsible decisions where the need to analyze problems, reflect, make decisions and take sensible actions [2]. One of the key elements that is argued in the body of this paper is to suggest that one aspect of an institution's academic maturity is what is presented to the public in the form of the academic bulletin of what is and what is not taught in the curriculum. While it cannot be stated with any certainty that academic maturity is predicted through the institution's academic bulletin, it is our opinion that an examination of a chiropractic college's academic bulletin provides a clue to an institution's academic maturity.

In the process of forming an opinion on the academic maturity of the chiropractic profession one important source of information to inform that opinion comes in review of the college catalog or academic bulletin. The academic catalog presents the curriculum. The curriculum is essentially the exposition of what domains of knowledge are taught in the classroom and what domains are evaluated in assuring student competence [2].

College catalogs and academic bulletins are designed by institutions as a means of communicating to students, parents and the public, as well as the faculty and administration of the college. A college catalog contains degree requirements for all programs of study that a particular institution of higher learning offers. Of main importance is that all institutions use the college catalog as the evolving document of authority for all students for academic institutions in the United States. However, as a document of authority, it serves as the first measure by which an outsider can assess the academic program. It is a means to communicate the quality of the program to the outside world.

In the chiropractic profession, one of the most controversial topics has to do with the concept of the subluxation. Other topics such as chiropractic philosophy and certain manipulative techniques certainly deserve mention and play a key role in the subluxation debate. Yet there is little doubt that there is much division within the chiropractic profession caused by the use of the term subluxation which is documented in the literature as a theoretical construct [3-5]. To date there has not been a study by which to assess the frequency of the usage of the term subluxation amongst the chiropractic institutions' curricula. Given the controversial nature of the subluxation construct knowledge about the prevalence of usage of the term in educational institutions is valuable in developing profession wide policy concerning the subluxation construct. The purpose of this study is to examine the most current North American English-language chiropractic college catalogs and academic bulletins to determine the prevalence of usage of subluxation in the academic curriculum.

## Methodology

Curricula from English-language chiropractic colleges in the United States and Canada were studied. The chiropractic colleges were identified from the Association of Chiropractic Colleges accreditation list [6]and their websites were accessed for availability of an online academic catalog/bulletin. The chiropractic schools that were accessed are seen in Table 1.

**Table 1:** Accredited English-Language Chiropractic Colleges

Cleveland Chiropractic College (Overland Park, KS and Los Angeles, CA)

Life University (Marietta, GA)

Canadian Memorial Chiropractic College (Toronto, Canada)

D'Youville College (Buffalo, NY)

New York Chiropractic College (Seneca Falls, NY)

University of Bridgeport (Bridgeport, CT)

Life Chiropractic College-West (Hayward, CA)

Palmer Chiropractic College (Davenport, IA, San Lorenzo, CA, Port Orange, FL)

Texas Chiropractic College (Pasadena, TX)

National University of Health Sciences (Lombard, IL)

Logan University (Chesterfield, MO)

Northwestern Health Sciences University (Minneapolis, MN)

Sherman College of Chiropractic (Spartanburg, SC)

Western States Chiropractic College (Portland, OR)

Parker College of Chiropractic (Dallas, TX)

Southern California University of Health Sciences (Whittier, CA)

Each college website was searched for the most recent academic catalogs, college catalogs, or academic bulletins. Only those catalogs that referenced either 2009 or 2010 academic years were used. Each of the schools provided access to an Adobe Acrobat PDF (portable document format) version. Every one of the chiropractic school's catalogs detailed a list of what constituted the DCP curriculum. This was noted as "schedule of courses." Using the search feature of the Adobe Acrobat reader program the word "subluxation" was searched. Categories were developed for the usage of the term and are shown in Table 2.

**Table 2:** Categories of methodology

Total times mentioned

Subluxation mentioned in a course description

Subluxation mentioned in a course title description

Subluxation mentioned in a technique course description

Subluxation mentioned in a chiropractic principles and practice course description

We determined how often "subluxation mentioned in a course description." However, if a specific course description mentioned the term subluxation more than once or if the title of the course had the term subluxation and the course description made mention to subluxation it was counted just once towards the total "subluxation mentioned in a course description". This was done to prevent double counting.

The percentage of courses that mention the term subluxation was calculated by dividing the number of courses mentioning the term subluxation by the number of courses in the DCP. For example, if a DCP had 10 courses in its curriculum and the subluxation term was mentioned once in all of those courses then the percentage would be 10% of the curriculum.

An aggregate mean for all DCPs was calculated for "total times mentioned" and "subluxation in a course." An aggregate mean percentage for all DCPs was calculated from the percentage each school used the term subluxation in all of its courses.

Several colleges used the same catalog (Cleveland Chiropractic College-Kansas City and Cleveland Chiropractic College-Los Angeles; Palmer College of Chiropractic has three campuses: Davenport, Iowa; Port Orange, Florida; and San Jose, California). Each school with multiple campus sites were studied as one institution. The three Palmer schools used one catalog but listed three individual DCP curriculums. The three Palmer school's standard curriculums for the DCP were examined individually. However, the Palmer school system's catalog, as a whole, was examined to determine subluxation usage.

## Results

Sixteen chiropractic college academic catalogs were studied. A total of eighteen English-language chiropractic school curricula in North America were assessed. The schools to prominently utilize the term subluxation overall were Palmer Chiropractic College (55 occurrences) and Sherman College of Chiropractic (37 occurrences) followed by Life University (Marietta, GA) (22 occurrences) and Life University -West (14 occurrences) (Table 3). Three schools did not mention the term: Southern California University of Health Sciences, Canadian Memorial Chiropractic College and National University of Health Sciences. An examination of the Palmer College catalog offered a challenge to the findings. Palmer College has three campuses with three different curricula. For example, Palmer College-Florida campus has a curriculum based on three categories: structure, function and care. It was determined that philosophy and technique were incorporated into these headings thus not counted as a standalone philosophy or technique course unless otherwise stated or identifiable as such.

**Table 3:** The usage of the term subluxation in the Doctor of Chiropractic Program (DCP)

School	Subluxation	Subluxation in courses	Total courses*	%subluxation/curriculum
Bridgeport	6	1	74	1.35

Cleveland	6	4	79	5.06

D'Youville	6	2	46	4.34

Life (GA)	28	24	146	16.44

Life (CA)	14	8	92	8.70

Logan	12	5	96	5.21

Northwestern	7	2	85	2.35

New York	5	3	72	4.16

Parker	16	6	74	8.11

Sherman	53	11	86	12.80

Texas	2	2	66	3.03

Western States	6	5	104	4.80

Palmer (IA)	55	6	72	8.33

Palmer (CA)	55**	5	78	6.41

Palmer (FL)	55**	10	44	22.72

National University	0	0	75	0.00

Southern California	0	0	67	0.00

Canadian Memorial	0	0	58	0.00

*Standard curriculum for DCP

**Not calculated in mean for "subluxation total"

The percentage of times that schools use the term subluxation can be seen in Table 3. Palmer (Port Orange, FL) led the schools with using the word subluxation in 22.72% (10/44) of the courses in their curriculum, whereas Life University (Marietta, GA) and Sherman College of Chiropractic followed with 16.44% (24/146) and 12.80% (11/186) of their courses include the word subluxation, respectively.

The aggregate mean number of appearances of the term subluxation in college catalogs across all DCP's studied is 13.50 (ranging from 0 to 55). The aggregate average number of courses or individual subjects that the term subluxation appears in across the entire chiropractic educational system curricula was a mean of 5.22. For several schools, the greatest use of the word subluxation was found in the non-curricular portions of the respective examined academic catalogs. For example, Sherman College of Chiropractic made mention of the term over 40 times throughout their college catalog apart from the curriculum. Likewise, Palmer College as a catalog representing three differing curricula mentioned the word subluxation over 30 times in the non-curricular portion of the respective institution's academic catalog.

Usage of the word subluxation can be seen in specific courses. Table 4 quantifies the usage of the word subluxation in course title, technique course and in a philosophy course. Very few chiropractic colleges have the term subluxation in the title of individual courses or subjects. However, subluxation was mentioned in technique courses and philosophy courses. Overall, as an average for all schools, 2.66 technique courses used the term subluxation in their descriptions. As for philosophy courses, all schools combined had an average of 1.66 courses of their curricula referred to subluxation in the philosophy course descriptions.

**Table 4:** Specific usage of term subluxation in the DCP

School	course title	technique course	philosophy course
Bridgeport	1	0	1

Cleveland	0	2	2

D'Youville	0	2	0

Life (GA)	1	14	1

Life (CA)	1	5	1

Logan	0	2	3

Northwestern	0	1	0

New York	0	1	2

Parker	0	4	1

Sherman	1	6	1

Texas	0	0	2

Western States	0	3	2

Palmer (IA)	0	4	2

Palmer (CA)	0	2	3

Palmer (FL)	4	2	0

National University	0	0	0

Southern California	0	0	0

Canadian Memorial	0	0	0

	x = 0.44	x = 2.66	x = 1.16

## Discussion

Academic catalogs reflect the general content of education at the time they are posted. Accreditation agencies as well as outside interests look to the college academic catalog as one of the sources of information concerning the academic policies and content. An academic catalog often contains rules and policies that affect students, descriptions of courses along with a list of the faculty and administration of the institution. Although academic catalogs are considered evolving documents and all college catalogs have disclaimers informing people that items can change without notice. Nevertheless, the catalogs serve as a reflection of the program as a whole and serve to provide the outward face of the program to the public. This study focused on the use of the term subluxation as an element of those programs and what they represent. It also examined the entire curriculum of each chiropractic school to determine where the term subluxation appears in the course descriptions and elsewhere in the catalog. It is acknowledged that when a course description includes a particular topic this does not guarantee that it will be discussed. Similarly, the lack of mention of a topic does not ensure the topic is not included in the actual course content.

### The subluxation construct in the DCP curriculum

Murphy et al [7] noted nine areas of reform that were essential to the mainstreaming of the chiropractic profession. One of these areas included educational reform, in particular the need to discontinue the perpetuation of dogma and unfounded claims. The most frequently encountered unsubstantiated claim related to the putative clinical meaningfulness of subluxation [8]. Keating et al have found that the subluxation construct lacks adequate evidence as a theoretical construct and appears to be more of a dogma [3]. Our study found that 50% of English-language doctor of chiropractic educational programs in Canada and the United States continue to teach this dogma. This contrasts with the chiropractic programs in the United Kingdom which do not teach about the subluxation or only do in a historical context [9].

One might argue that a historical presentation of the subluxation may be all we are finding references to in our search of the DCP curricula. This would be a reasonable argument if the subluxation was mentioned in only one course in a DCP program or if it was only in what was described as a philosophy course. This, in fact, only occurred at two colleges (Texas Chiropractic College and the University of Bridgeport College of Chiropractic), however, with an aggregate average of 13.5 appearances and many of those in technique classes. It seems unlikely that this is purely to explain the history of term in the chiropractic profession.

Faculty and alumni resistance may play a part in the continued teaching about the subluxation. Lawrence [10] noted that getting faculty to change is akin to trying to move an iceberg with a toothpick. The recent uproar about the U.S. Council on Chiropractic Education's new accreditation standards [11,12] and General Chiropractic Council of the UK's position on the subluxation [13,14] shows how much resistance there is to dropping the term. However, other facts such as the committee structure and internal academic policy regarding course modification may be factors as well. The input of the alumni of a college may also prevent change. Many alumni continue to use the term [15,16] and thus might desire future generations of chiropractors to be trained as they were.

### Evidence-based medicine

Since the concept of subluxation lacks a sufficient evidence base to support it as a real clinical entity, [3] continuing to teach this is in conflict with the need for the DCPs to be evidence-based [5,17,18].

While this study did not delve into the concept of evidence-based practice, some schools have devoted coursework to the concept. An interesting finding not related to the study design was that one particular college catalog appears to teach evidence-based concepts yet continues to teach the subluxation construct. This dichotomy is worth further examination in future studies. Palmer-West presented one course entitled "Evidence-based chiropractic I" with a course description similar to what one would expect in such a course i.e. development of skills in critical thinking, literature search, critical evaluation and integration of evidence [19]. However, this same curriculum offered in its "Chiropractic Philosophy & Practice II" course a description denoting concepts of subluxation and its mechanism of production and dysfunction [19].

Four chiropractic programs studied have received grants from the National Center for Complimentary and Alternative Medicine (a part of the U.S. National Institutes of Health) to improve the teaching of evidence base practice in their respective chiropractic curricula. Only one of those four does not include the subluxation in their curriculum (National University of Health Sciences). Future research should determine what affect, if any, these research grants have on the prevalence of the subluxation construct in these DCPs.

We believe that for the chiropractic education curriculum to become truly evidence-based it will have to make changes that reflect a current state of the knowledge base. This means that a certain number of unsupported beliefs and theories of the past will, of necessity, have to be abandoned. This will be the hallmark of a profession prepared to change as science and evidence changes and not of a craft group that follows an ideology espoused by its founder or guru.

McDonald et al [16] reported that over 88% of chiropractors surveyed favored retaining the term vertebral subluxation complex. Smith and Carber [15] found that over 70% of chiropractors reported that subluxation is important to their clinical decisions and guides their clinical care of patients. McDonald et al [16] reported that a strong majority (over 75%) of their surveyed chiropractors believed that subluxation was a significant contributing factor to 50% or more of visceral disorders. Despite this finding, Smith and Carber's research [15] demonstrated that most of their surveyed chiropractors seemed to believe that a subluxation-based clinical approach may be of limited utility for addressing visceral disorders, and greatly favored non-subluxation-based clinical approaches for such conditions. The findings of McDonald et al [16]and Smith and Carber's [15] support the notion that the chiropractic curriculum may be producing graduates who are using the subluxation construct as fact or readily accept the notion after course completion. Until and unless sound research published in credible journals demonstrates the existence and reliable identification of vertebral subluxation, and vertebral subluxation is found to be an important public health problem, society at large will be skeptical and not care about its correction [5].

There is an obvious disconnect between those who are skeptical and critical of the subluxation and those practicing in the field who adhere to it. The few in academic circles that may be highly critical of the subluxation construct appear to have had little influence on curtailing its use both inside and outside the chiropractic academy. Alumni who favor the term may be influential in this matter. This should not be surprising given that field practitioners hold in much disdain those who dedicate themselves to an academic career [10]. Nonetheless, faculties do have influence on curricular matters and the failure to curtail the use of subluxation in the chiropractic curriculum would undoubtedly indicate to critics that the faculty of chiropractic colleges is more than willing to embrace an unscientific concept such as subluxation. Such failure of faculty to be instrumental in curricular changes indicates that they themselves are not scientifically-minded or that college administration is unwilling to allow faculty the necessary steps to make progress in matters related to the curriculum. No matter who may be at fault in the curricular propagation of the subluxation construct, the lack of change may indicate that chiropractic, as an academic enterprise is willing to embrace scientifically unsubstantiated concepts. Using the slippery slope argument, if one area of the curriculum or even mission of the institution has one unsubstantiated construct such as subluxation, how many more unsubstantiated ideas, concepts, constructs are being perpetuated?

### The accreditation process

College catalogs are the one of the first lines of evidence to accreditation agencies. Three chiropractic schools made the decision to not make mention of the subluxation construct. What is perplexing about this aspect is found in the documents of the Council of Chiropractic Education (CCE) itself. The new CCE Accreditation Standards do not mention subluxation at all [20] and the new Manual of Policies only mentions the term one time in the meta-competencies:

*Performing case-appropriate physical examinations that include evaluations of body regions and organ systems, including the spine and any subluxation/neuro-biomechanical dysfunction, that assist the clinician in developing the clinical diagnosis(es) *[21].

The older Standards for Chiropractic Programs and Requirements for Institutional Status mentioned the term six times [22]. We wonder if this change in the accrediting agency's use of the term will result in a corresponding change at the DCP level. Thus, we anticipate replicating this study in a few years to determine what changes in DCP programs maybe seen after implementation of these changes in 2012.

### Chiropractic philosophy in the DCP curriculum

The philosophical basis of chiropractic is another topic of considerable importance. At the root of the chiropractic philosophical system and the connection to subluxation is the nervous system. One of the colleges' examined noted: "Our purpose as Doctor's of Chiropractic is to locate and correct any interference to the system in the body that controls and coordinates all functions--the nervous system"[23]. This same school utilized the subluxation term in two of its biochemistry courses. Since there is no scientific evidence to support subluxation at the biochemical level why is such a connection made in a biochemistry course? The reason this is relevant is that some chiropractors have compared chiropractic education to medical education. When an entity such as subluxation which has such little evidence to support its existence is portrayed as having an effect upon human biochemistry it gives the appearance that the dogmatic character of subluxation beliefs [3] has permeated into core scientific courses in the chiropractic program.

## Conclusion

The concept of the subluxation in chiropractic is a controversial subject with a paucity of evidence. With the exception of three schools, all English-language DCPs in North America mention the concept of the subluxation either in course titles or descriptions and/or in their respective missions. Despite the lack of evidence for the subluxation construct, it appears to be very much a key part of chiropractic education.

Some schools may state that they are not subluxation-focused or heavily engaged in the teaching of subluxation. Nonetheless, most schools continue to teach about the subluxation in what seems to be more than just a historical context. We believe that this puts the profession in an awkward position because the skeptic and/or critic of subluxation can point to chiropractic education as outdated and unscientific. Chiropractic education will have to address this issue if the chiropractic educational enterprise wishes to become scientifically competitive with other healthcare sciences and produce graduates who are critical thinkers prepared as the evidence changes to change their practice and throughout their careers. Future research should determine if changes in regulation and research change the prevalence of the use of the term subluxation in chiropractic curricula.

## Competing interests

The authors declare that no funding was received for completion of this review. TAM claims no conflict of interest. SMP is a full-time faculty member and on the post-graduate faculty of another of the institutions whose catalog was evaluated in this study. SMP claims no other conflict of interest.

## Authors' contributions

TAM extracted the raw data. TAM and SMP analyzed the data. TAM and SMP drafted the manuscript. All authors read and approved the final manuscript
